# Decision Support Systems in HF based on Deep Learning Technologies

**DOI:** 10.1007/s11897-022-00540-7

**Published:** 2022-02-10

**Authors:** Marco Penso, Sarah Solbiati, Sara Moccia, Enrico G. Caiani

**Affiliations:** 1 Department of Electronics, Information and Biomedical Engineering, Politecnico Di Milano, P.zza L. da Vinci 32, 20133 Milan, Italy; 2 Centro Cardiologico Monzino IRCCS, Milan, Italy; 3 Institute of Electronics, Information Engineering and Telecommunications (IEIIT), Italian National Research Council (CNR), Milan, Italy; 4 The BioRobotics Institute, Department of Excellence in Robotics and AI, Scuola Superiore Sant’Anna, Pisa, Italy

**Keywords:** Deep learning, Heart failure, Artificial intelligence, Prognosis, Diagnosis, Readmission

## Abstract

**Purpose of Review:**

Application of deep learning (DL) is growing in the last years, especially in the healthcare domain. This review presents the current state of DL techniques applied to electronic health record structured data, physiological signals, and imaging modalities for the management of heart failure (HF), focusing in particular on diagnosis, prognosis, and re-hospitalization risk, to explore the level of maturity of DL in this field.

**Recent Findings:**

DL allows a better integration of different data sources to distillate more accurate outcomes in HF patients, thus resulting in better performance when compared to conventional evaluation methods. While applications in image and signal processing for HF diagnosis have reached very high performance, the application of DL to electronic health records and its multisource data for prediction could still be improved, despite the already promising results.

**Summary:**

Embracing the current big data era, DL can improve performance compared to conventional techniques and machine learning approaches. DL algorithms have potential to provide more efficient care and improve outcomes of HF patients, although further investigations are needed to overcome current limitations, including results generalizability and transparency and explicability of the evidences supporting the process.

## Introduction

Heart failure (HF) represents a severe condition affecting approximately 2% of the adult worldwide population, thus counting around 36 million of individuals globally; it consists of a chronic and progressive syndrome characterized by structural or functional cardiac dysfunctions with reduced (HFrEF; < 40%) or preserved (HFpEF; ≥ 50%) left ventricular ejection fraction [1, 2, 3, 4]. HF represents the most rapidly growing cardiovascular disorder globally. Its pathological spectrum involves numerous symptoms able to greatly affect the patient’s quality of life, as dyspnea, fatigue, and poor exercise tolerance, leading to frequent hospitalizations and shortened life expectancy [2, 3].

**Fig. 1:**
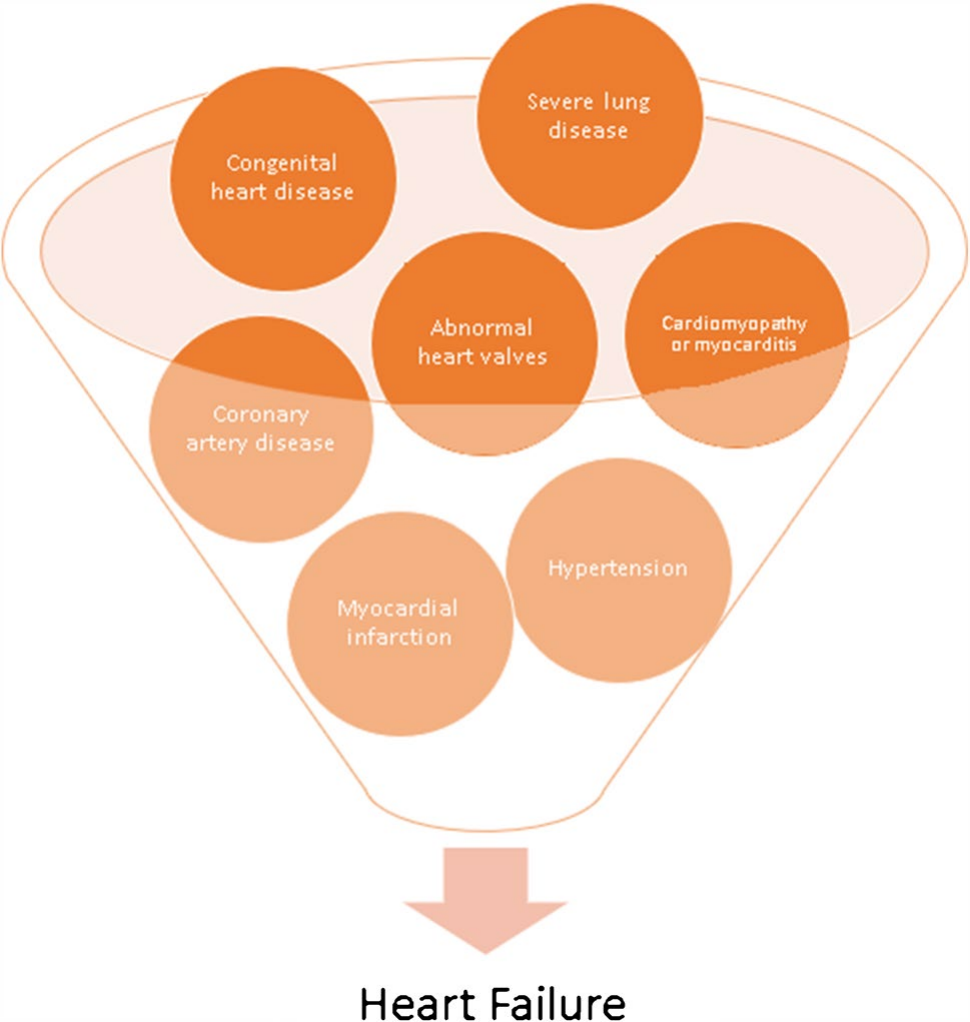
Schematization of the different pathological conditions that may lead to heart failure

Cardiac conditions and causes of death vary in the HF population, and although the main underlying causes of this syndrome have been identified, including coronary artery disease, valvular heart disease, hypertension, cardiomyopathies and other (Fig. 1), the prevalence of HF is expected to increase, accounting for a substantial burden to the healthcare system [4, 5]: specifically, due to the ageing population, treatment costs relevant to HF are expected to double by 2030 [6].

Despite advancements in clinical management, surgical procedures, and medical devices in the treatment of all causes associated with HF, significant challenges still persist in current treatments [5], and HF remains one of the main global health concerns [6]. Modeling the driving factors of HF for achieving high prediction accuracy in both diagnosis and prognosis is still an unmet medical need. Accordingly, there is the need for novel approaches to optimize the management of this chronic disease, to improve clinical decision-making, and to ultimately reduce related healthcare expenditures. As HF has been recognized as a heterogeneous multifactorial syndrome, improvement in the assessment and management of HF patients requires to integrate data obtained by different sources (e.g., laboratory, echocardiographic and morphologic data) and to handle the complex interplay of various symptoms and comorbidities (both cardiovascular and non-cardiovascular) involved in the HF pathology.

Healthcare is undergoing a new era characterized by the availability of a massive amount of biomedical data, which necessarily opens to new opportunities. The advancement of big data solutions within the healthcare system has allowed to store and manage huge amount of data with the aim to develop new disease risk assessment tools and prediction models, but exploiting in clinical practice these advances leads to unprecedented challenges regarding data analysis and interpretation, as well as many difficulties related to heterogeneity, quality, and integrity of the healthcare data [7].

**Fig. 2:**
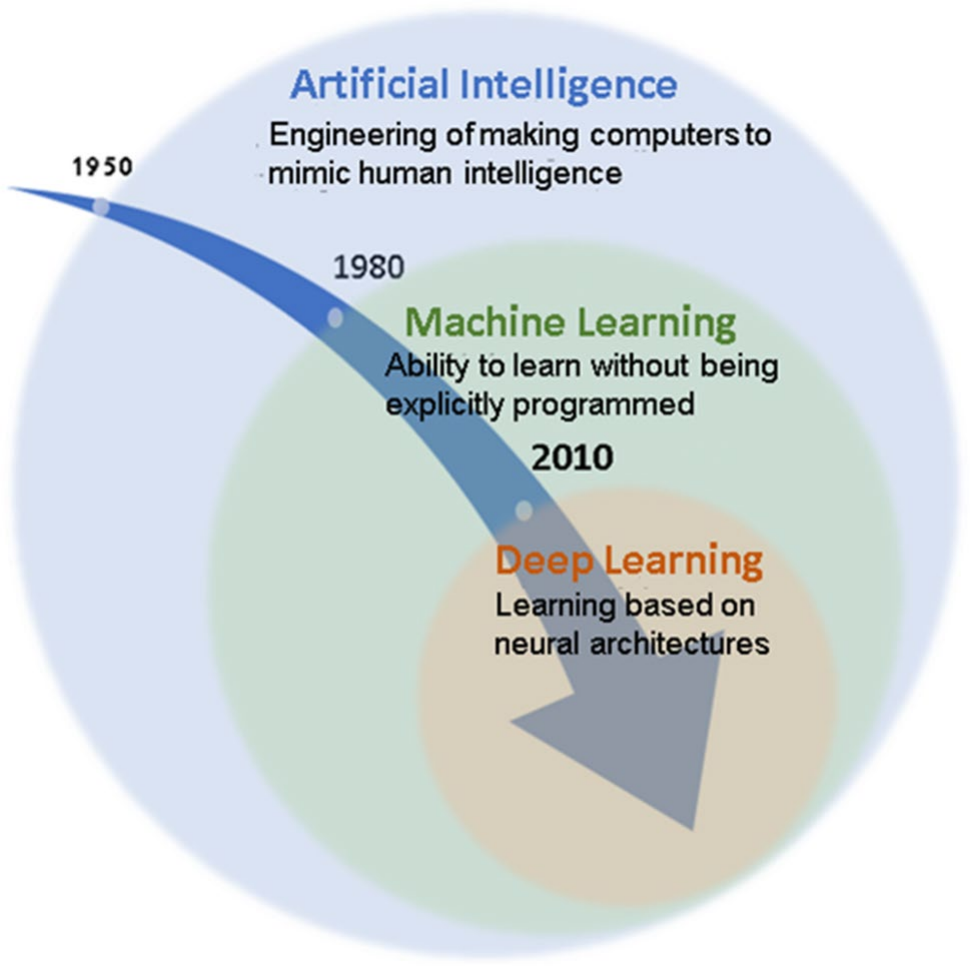
Evolution of artificial intelligence and its main components, in which deep learning represents a subset of machine learning methods

Machine learning (ML) and deep learning (DL) methods, as a branch of artificial intelligence (Fig. 2), have experienced a rapid growth over the past few years achieving state-of-the-art performance in various domains, including medical imaging, diagnosis, and prognosis [8, 9, 10]. DL is a subfield of ML and represents a family of algorithms that can be used to learn complex and highly predictive patterns that generally remain unexplored using conventional statistic approaches. In contrast to ML, learning solutions based on DL do not require to design a priori feature extractors from which the learning algorithm detects patterns [11]. In this way, the algorithm is free to learn by itself, automatically defining the features to be considered and the patterns to be searched in order to perform classification or prediction. An additional advantage for DL solutions is the possibility to integrate different structured and unstructured data types as input, which is particularly relevant considering the typical heterogeneity of the healthcare data [11].

In this context, the aim of this review is to provide key concepts for DL that clinicians need to be familiar, and to give an overview of the current advancements of DL research in several clinical applications for the treatment of HF patients.

The paper is organized as follows: in the “Deep Learning: Key Concepts for Clinicians” section, the fundamental concepts of DL along with a presentation of common DL models used in cardiology are given; in the “Deep Learning in HF Diagnosis” and “Deep Learning in HF Prognosis (End of Hospitalization)” sections, an overview of recent DL approaches for HF diagnosis and prognosis, respectively, are described. Thereafter, in the “Deep Learning for Predicting HF Readmission: from EHR to Home Monitoring” section, a review of DL solutions for predicting HF hospital readmission is presented, and in the “Challenges for Deep Learning” section, current challenges for DL solutions are discussed. Finally, in the “Conclusion” section, conclusions are drawn with a focus on the future directions for DL applied to the treatment of HF patients.

## Deep Learning: Key Concepts for Clinicians

As previously introduced, DL constitutes a subset of ML methods building on the foundations of neural networks, thus trying to mirror the way the human brain processes data (in particular, its learning ability). In contrast to conventional ML methods, requiring human interaction to define which features in the available data have to be considered important for the solution of the classification/prediction problem, DL is meant to learn by itself how to extract knowledge from the data, without being explicitly programmed [12]. In other words, DL automatically extracts the features from the data that are considered important to solve the given task, thus removing the need to select them beforehand, and without involving prior knowledge to explain the observed variability in the data. These fundamental characteristics have generated enthusiasm about the potential of DL to solve problems beyond the human capability, and in recent years, the utilization of DL approaches, that have demonstrated to perform at human-level efficiency and in certain tasks even with higher performance than expert clinicians [12, 13, 14], has surpassed that of ML. The main reasons behind this success, compared to learning algorithms based on hand-designed methods, have to be found in the increasing available computational power, in the larger availability of data, and in the rapid algorithms’ development. Indeed, in few years, DL has been able to show its potential in defining new opportunities for improving therapies and treatment, in performing early diagnosis and in reducing the length of hospitalization.

Within DL, artificial neural network (ANN) is an information-processing system, whose structure and functionality simulate the nervous system and the human brain [15]. The main element is the neuron, a simple processing unit, that sends information to other neurons through action potentials, and working in parallel these neurons define a layer. As the brain processes information through multiple stages of transformation, similarly the ANN is characterized by multiple layers of neurons, in order to achieve learning capability [15]. Thanks to the fact that each layer performs a nonlinear mapping based on the previous layer’s output, this allows the network to learn via progressive levels of information abstraction [15, 16]. This ability to learn features at multiple level of abstraction allows the network to learning complex functions, without depending on manually developed features.

The most common type of ANN is the convolutional neural network (CNN), which was inspired by the structure of the human visual system. A CNN can be considered an ANN with many identical copies of neurons in its layers, thus utilizing the local relationship within the data to extract spatial features. This allows the network to increase the number of neurons, and hence its computational power, while keeping the number of learnable parameters relatively small. CNNs are designed to process arrays of data: in 1D as signals (e.g., electrocardiographic, audio, or textual data); in 2D as images; and in 3D as video or volumetric data [16]. Considering an image as input, the first layers of the CNN are associated in learning how to recognize basic lines and curves; moving more deeply, the following layers apprehend shapes and blobs, while in the last layers the ability to classify increasingly complex objects within the image is reached. One of the most popular CNN architecture for medical image analysis is represented by the U-Net [17], that has shown impressive performance, even with a scarce amount of training samples.

Recurrent neural network (RNN) represents another class of ANN, able to recognize patterns in temporal or sequential data [15, 18]. In contrast to common ANN, where the inputs are independent from each other, the main characteristic of RNN is the ability to remember information from prior inputs to generate the current output. In this way, the output of RNN depends on the current input and on all the previous elements of the sequence, where each neuron acts as a memory cell while computing operations. While CNNs are suitable for handling spatial information, RNNs are more suitable for handling temporal or sequential information. For example, given a sequence of frames, a RNN takes the first frame and makes a prediction; the prediction of the following frame is conditioned by the information obtained on the previous frame. Two popular architectures in the RNN family are the gated recurrent units (GRU) and the long short-term memory (LSTM), designed to process information over extended time [19, 21].

More recently, generative adversarial network (GAN) has been introduced in the field of DL, and has increasingly been used in several medical image analyses applications, such as denoizing, reconstruction, segmentation, synthetization, classification, and image-to-image translation. Thanks to its impressive performance, GAN has gained a lot of attention: as its name suggests, unlike conventional ANN, GAN consists of two networks, known as generator and discriminator, trained in an adversarial way [22]. While the generator tries to generate new data, the discriminator learns to distinguish the synthetic data from the real ones. The goal of the discriminator is to force the generator in improving its performance in learning to generate a more realistic data distribution, with the aim of deceiving the discriminator.

For all these methods, the common development procedure starts from the availability of a labeled (i.e., gold standard) dataset, where each data is classified by the expert binary (i.e., healthy and pathologic) or multi-class classification. This dataset is divided into training, validation, and testing sub-datasets: the training dataset is used to automatically generate the features able to reach the expected goal, compared to the gold standard labels in terms of specific, sensitivity, and accuracy, often summarized in the area under the receiver operating characteristics (ROC) curve. The validation is used to further tune other parameters in the network in the attempt to further optimize its performance. Finally, the real performance is computed by testing the developed network on the testing dataset.

## Deep Learning in HF Diagnosis

An early diagnosis of HF may reduce patients’ mortality and morbidity. Consequently, wide efforts have been put in the research to develop algorithms to support clinicians in early diagnosing HF. HF diagnosis may be achieved through the analysis of electrocardiography (ECG), as well as medical images, mainly acquired through magnetic resonance (MRI) and ultrasound (US) images. Furthermore, electronic health records (EHRs, also known as medical records) can be used to this purpose. Early approaches mainly exploited model-based algorithms, while more recently data-driven algorithms (i.e., ML and DL) have shown interesting results given their ability to tackle the complexity and variability of clinical data acquired from subjects at risk of developing HF. The papers surveyed in this section are summarized in Table 1, where the publication date, data source, aim, algorithm, and dataset size are specified.

**Table 1:** Summary of recent studies exploiting deep learning algorithms for diagnosis of HF

Author	Year	Outcome	Data source	Dataset	Algorithm	Results
Kwon et al. [21]	2019	HF (reduced or mid-range to reduced EF) identification	ECG (RR interval recordings)	55,163 ECG from 22,765 patients	ANNs	AUC = 0.889 for HF with reduced EF AUC = 0.850 for HF with mid-range to reduced EF
Çınar et al. [22]	2021	HF diagnosis	ECG (RR interval recordings)	162 subjects	CNNs and support vector machines	Accuracy = 0.97
Acharya et al. [23••]	2019	Congestive HF diagnosis	ECG (RR interval recordings)	140,000 ECG segments	CNNs	Accuracy = 0.99 Specificity = 0.99 Sensitivity = 0.99
Lih et al. [24•]	2020	Congestive HF diagnosis	ECG (RR interval recordings)	262 subjects	CNNs and long short-term memory	Accuracy = 0.98 Specificity = 0.98 Sensitivity = 0.99 Positive predictive value = 0.97
Wang et al. [25]	2019	Congestive HF diagnosis	ECG (RR interval recordings)	Five open datasets (BIDMC, NSR, FD, NSR-RR, and CHF-RR) for a total of 178 subjects	CNNs and long short-term memory	Heart rate variability accuracy on BIDMC, NSR, and FD = 0.99 Accuracy on CHF-RR and NSR-RR = 0.88
Lei et al. [26]	2021	Congestive HF diagnosis	ECG (RR interval recordings)	83 subjects	CNNs based on U-Net	AUC = 0.90 Accuracy = 0.89
Jahmunah et al. [27]	2021	Congestive HF diagnosis	Lead II ECG signals	262 subjects (92 healthy controls, 7 CAD, 148 MI, and 15 CHF)	CNNs and Gabor filtering	Accuracy = 0.99
Choi et al. [30••]	2017	HF diagnosis	EHRs	3884 HF + 28,903 control subjects	RNNs with gated recurrent units	AUC = 0.88 (18-month observation period)
Maragatham et al. [31]	2019	HF diagnosis	EHRs	4289 HF + 30,249 control subjects	RNNs with long short-term memory	AUC = 0.89
Rasmy et al. [32]	2018	Prediction of HF risk	EHRs	150,000 HF + 1,000,000 control subjects	RNN with long short-term memory	AUC = 0.82
Ma et al. [33•]	2019	HF diagnosis	EHRs	4925 HF	CNNs	Accuracy = 0.91
Gao et al. [29]	2020	HF diagnosis	Heart sounds	2543 subjects (42 HF reduced EF, 66 HF preserved EF, 2435 controls)	Gated recurrent unit model	Accuracy = 0.99
Lan et al. [34]	2019	Left ventricle segmentation for EF evaluation	MRI	45 volumes from 45 subjects (12 HF with ischemia, 12 HF without ischemia, 12 hypertrophy, 9 normal)	CNNs	DSC = 0.97
Baessler et al. [35]	2019	Diagnosis of HF myocarditis	MRI	71 subjects	k-Nearest neighbors	AUC = 0.85
Tabassian et al. [36•]	2018	Diagnosis of HF	Echocardiography stress test (strain parameters)	100 subjects (33 with HF preserved EF, 67 controls)	k-Nearest neighbors	Accuracy = 0.85 AUC = 0.89
Cikes et al. [37••]	2019	Phenogrouping a HF cohort	Echocardiographic data and clinical parameters	1106 HF subjects	Unsupervised ML algorithm (k-means clustering)	Four phenogroups were identified. Group 1 and 3 reported a 64% and 65% reduction in the risk of HF or death, respectively (hazard ratio 0.36, *p* = 0.001) after defibrillator treatment
Seah et al. [38]	2019	Diagnosis of congestive HF	Radiography	103,489 frontal chest radiographs in 46 712 patients	CNNs	AUC = 0.82

*CAD*, coronary artery disease; *MI*, myocardial infarction; *CHF*, congestive heart failure; *HF*, heart failure; *HERs*, electronic health records; *ECG*, electrocardiography; *MRI*, magnetic resonance images; *AUC*, area under the curve; *ANN*, artificial neural network; *CNNs*, convolutional neural networks; *EF*, ejection fraction; *RNNs*, recurrent neural networks; *DSC*, dice similarity coefficient; BIDMC: Beth Israel Deaconess Medical Center CHF database; NSR: normal synus rhythm; FD: Fantasia database

The widest literature in the field can be found for the processing of the ECG signal. In Kwon et al. [23••], a ML algorithm based on ANN was proposed for HF identification. The ANN processes both demographic and ECG features, achieving an area under the receiver operating characteristics curve (AUC) of 0.89. In Çınar et al. [24•], a more advanced algorithm based on CNNs was used to automatically extract features from the ECG spectrogram. The features were classified using support vector machines (SVMs), achieving an accuracy of 0.97.

A fully DL-based pipeline was proposed by Acharya et al. [25] which exploits a CNN to automatically extract relevant features from the ECG signals and performs an early diagnosis of HF. The pipeline allows to perform end-to-end training, lowering the training time, with an achieved accuracy of 0.99. Lih et al. [26] coupled CNNs with long short-term memory (LSTM) to keep into account the temporal information naturally encoded into the ECG, obtaining an accuracy of 0.98. A similar approach was used in [27], which further included an inception module in the CNN to allow multi-scale analysis, thus achieving an accuracy of 0.99.

A more complex CNN architecture, based on U-Net, was proposed in [28], where residual blocks were exploited to perform a more accurate feature extraction and classification, reaching an AUC of 0.90. Residual block adds the output of a previous layer to the output of the following layer to extract some additional spatial information. In [29], the first layer of a custom CNN was replaced by Gabor filters to lower the training complexity while extracting relevant high-frequency ECG features, with a reported accuracy of 0.99. Gabor filters are linear filters used for texture analysis and feature extraction, which have been shown excellent localization properties both in spatial and frequency domain, simulating the receptive fields of the human visual system [30••].

As regards other signals, a recent work [29] investigated the possibility to diagnose HF from heart sounds, where logistics regression and gated recurrent units were used to identify the presence of HF: despite the promising results (accuracy = 0.99), more research is still required in this field.

In the last decades, also the analysis of EHRs to perform HF diagnosis has been receiving attention, thanks to the large availability of digitalized data, as well as to the development of more and more accurate ML/DL algorithms. Choi et al. [30••] proposed a milestone paper on the use of recurrent networks for early detecting HF onset (achieving an AUC of 0.88) and, based on it, several works have been published following a similar paradigm. Examples include [31], that used LSTM to process time-stamped EHRs containing medicinal information achieving an AUC of 0.89, and [32], that classified a large variety of features (e.g., demographic, procedural, medicinal features) with LSTM achieving an AUC of 0.82. A more advanced approach was proposed by Ma et al. [33•] that built an embedding from the EHR using CNNs and attention mechanisms, where the embedding was classified with a custom-built prediction model achieving an accuracy of 0.91.

With the goal of predicting HF onset, applications of ML and DL to the field of image processing have been also proposed. In particular, several papers have focused on MRI imaging: in [34], a DL algorithm for the automatic segmentation of the left ventricle as a prior to evaluate the cardiac function in HF patients was proposed, where a Dice similarity coefficient of 0.97 was achieved. A ML method based on k-nearest neighbors was used in [35] to perform texture analysis of myocardial maps and identify early symptoms of HF, achieving an AUC of 0.85.

Besides MRI, other works focused on echocardiographic imaging: Tabassian et al. [36•] analyzed spatiotemporal patterns of echocardiographic deformation curves using k-nearest neighbors with an accuracy of 0.89, while Cikes et al. [37••] evaluated echocardiographic patterns using k-means clustering to identify pathogroups in patients with HF. An interesting attempt of predicting HF markers from chest radiographs with DL was performed by Seah et al. [38] obtaining promising results (AUC = 0.82), but more research is still needed to understand the potentiality of DL in processing chest radiographs for HF diagnosis.

## Deep Learning in HF Prognosis (End of Hospitalization)

Several studies have used DL to predict different outcomes in HF patients [39, 40•]. Specifically, the measured outcomes that were studied include mortality, hospitalizations, readmissions, risk prediction, need for mechanical circulatory support, heart transplantation, and treatment effect (Table 2). The general process of DL techniques regarding HF prognosis is based on data obtained through the EHRs that might include demographic information, treatment and medication, laboratory results, ECG, and echocardiographic findings before and during hospital stay. Wang et al. [41••] applied ANN for early detection of patients’ HF death in three observation windows (i.e., in-hospital, 1-month and 1-year mortality), studying 10,203 in-patient EHRs. It is noteworthy the introduction of a focal loss function [42] into the proposed framework, to deal with the imbalanced class problem, and a feature rearrangement layer to improve feature representation of the convolutional network. The proposed ANN provided an AUC in predicting mortality of 0.904 (in-hospital), 0.891 (1-month observation), and 0.887 (1-year observation).

**Table 2:** Summary of recent studies exploiting deep learning algorithms in HF prognosis

Author	Year	Outcome	Data source	Dataset	Algorithm	Results
Medved et al. [47]	2018	Survival prediction after heart transplantation	EHRs	27,705 patients	ANNs	Reduction of 12% for ROC and 10% for C-index by using deep learning technique
Wang et al. [41••]	2020	Mortality prediction	EHRs	10,203 patients	CNNs	AUC in-hospital 0.904, 1-month 0.891, 1-year 0.887
Golas et al. [48•]	2018	Readmission prediction	EHRs	11,510 patients	Deep unified networks	AUC 0.705
Kwon et al. [43]	2019	Mortality prediction	EHRs	6924 patients	ANNs	AUC in-hospital 0.880, 12-month 0.782, 36-year 0.813
Lewis et al. [50•]	2021	Preventable hospitalizations, emergency department and costs	Clinical history	93,260 patients	ANNs	AUC for deep learning were 0.778, 0.681, and 0.727, respectively
Ashfaq et al. [39]	2019	Readmission prediction	EHRs	7655 patients	RNNs with long short-term memory	AUC 0.77
Chu et al. [49•]	2020	Treatment effect prediction	EHRs	736 patients	GAN	AUC 0.688
Kwon et al. [46•]	2019	In-hospital mortality	Clinical + echocardiography	760 HF	ANNs	AUC 0.913
Li et al. [51••]	2020	Risk prediction	EHRs	554 HF + 1662 controls	RNNs	RNN outperforms the state-of-the-art approaches by approximately 1.5%
Pandey et al. [59]	2021	Phenotyping diastolic dysfunction in HF with preserved ejection fraction	Echocardiography	1242 patients	ANNs	AUC 0.88
Hearn et al. [53]	2018	Clinical deterioration	Cardiopulmonary exercise test data	1156 HF	ANNs	AUC 0.842
Lu et al. [52••]	2021	Long-term trajectory prediction	EHRs	8093 HF	RNNs with gated recurrent units	AUC 0.863

*HF*, heart failure; *HERs*, electronic health records; *AUC*, area under the curve; *ANN*, artificial neural network; *CNNs*, convolutional neural networks; *RNNs*, recurrent neural networks; *GAN*, generative adversarial networks

Kwon et al. [43] used a DL-based model in a multicenter cohort of acute HF patients for predicting in-hospital mortality, and at 12 and 36 months, by integrating clinical and laboratory data. Training included 2165 patients, while validation was performed on 4759, reaching an AUC for predicting in-hospital and 12 and 36 months mortality of 0.880, 0.782, and 0.813, respectively. Overall, DL outperformed both the conventional Get with the Guidelines–Heart Failure (GWTG-HF) score, and the Meta-Analysis Global Group in Chronic Heart Failure (MAGGIC) score [44, 45], as well as other ML models. Since GWTG-HF and MAGGIC cannot be used for initial treatment or screening, Kwon et al. [46•] applied DL to predict in-hospital mortality only on echocardiographic data in 25,776 patients. In a subgroup analyses of HF, DL provided an AUC (0.913) higher than both MAGGIC (0.806) and GWTG-HF (0.783) scores. Medved et al. [47] compared the International Heart Transplantation Survival Algorithm (IHTSA) based on DL, with the Index for Mortality Prediction After Cardiac Transplantation (IMPACT), for predicting 1-year survival after heart transplantation. In 27,705 patients (5597 in the test cohort), DL exhibited an AUC of 0.654, with improved performance compared to the IMPACT model (AUC 0.608). Although IHTSA was designed to predict long-term survival, it showed better discrimination at 1-year mortality than IMPACT. Therefore, even though modest, these results are promising for DL techniques applications in clinical practice.

To model early HF readmission prediction, a deep unified network, an innovative architecture designed to avoid overfitting including both structured (i.e., demographics, clinical and laboratories results) and unstructured (physician notes and discharge summaries) data from EHRs of 11,510 patients, was applied [48•]. Obtaining an AUC of 0.705, the developed 30-day readmission model reported the best performance compared to logistic regression (LR) (0.664), gradient boosting (0.650), and maxout networks (0.695). In a novel study applying DL to EHRs for treatment effect prediction on 736 HF patients, the proposed generated GAN learning strategy outperformed benchmark models in terms of both accuracy (0.688) and AUC (0.654) [49•]. The DL treatment effect prediction model used two auto-encoders for learning features of both patient characteristics and treatments from EHRs. Specifically, the DL scheme could generate and discriminate the predicted treatments from the real ones so that highly representative features were extracted from the EHRs data [49•].

In [50•], 93,260 HF patients were analyzed to identify preventable outcomes, such as hospitalization and emergency department visits. Compared to ML and LR models, DL produced the highest AUC of 0.778 and 0.681, respectively. Remarkable was the effort of Li et al. [51••] in terms of interpreting DL models, by developing an interactive clinical risk prediction system based on RNN with an intuitive visualization design, increasing transparency to the information infrastructure, thus allowing visual interpretation of the prediction results. On 554 HF and 1662 control patients, the proposed DL model outperformed the state-of-the-art approaches by approximately 1.5%. Recently, Lu et al. [52••] proposed a DL approach to model long-term and short-term HF clinical trajectories on 8093 patient with congenital heart disease. The network outperformed various baseline models and was able to predict different types of patient trajectories (AUC 0.863). A separate study used DL to add prognostic value of data acquired from a cardiopulmonary exercise test (CPET) [53]. In another study involving 1156 HF patients, DL demonstrated little improvement compared to statistical model (AUC 0.842 vs. 0.837), while both were superior to CPET-risk score (AUC 0.759) [54].

Finally, ML and DL seem promising in identifying distinct patient subgroups with HFpEF using unsupervised learning to deliver more tailored clinical care. These techniques make it possible to learn from an input dataset without the need for training with labeled data (expected outputs). In fact, the model learns to draw inferences and identify significant features within the unlabeled data space, for the purposes of clustering or data reduction. The pathological development of HFpEF has been attributed to a complex interplay of cardiac and extracardiac dysfunctions [55, 56] leading to a marked phenotypic heterogeneity among patients of this population. This diversity highlights the fact that there is not a single pathological process underlying the observed dysfunction, thus affecting the targeted management plan. Recently, different studies made progress in clustering HFpEF patients by integrating multiple patient data, as step towards personalizing treatment and improving prognosis of the disease [57, 58]. Pandey et al. [59] analyzed 1242 HFpEF to predict high- and low-risk phenogroups and validated the network in 5 external cohorts. The DL approach showed higher AUC than the 2016 American Society of Echocardiography guideline–based left ventricular grades [60] for predicting elevated left ventricular filling pressure (0.883 vs. 0.676). Kaptein et al. [61] proposed an unsupervised learning approach to identify subgroups of patients with asymptomatic diastolic dysfunction, where three subgroups were identified. Similarly, in [62], a model-based clustering on clinical and echocardiogram variables in 320 HFpEF patients was applied, from which six phenogroups were derived. Although HFpEF remains a challenging clinical condition to manage, clustering patients with model-based learning using echocardiographic and EHRs data may provide better granularity with improved prognostic benefit for patients with HFpEF compared to the current clinical paradigm, thus creating phenotype clusters that are strongly linked to survival. This new approach may lead to improved personalized care pathways for treating patients with HF.

## Deep Learning for Predicting HF Readmission: from EHR to Home Monitoring

The significantly high rate of readmissions in hospital after HF, with 61.3% of the patients being readmitted for HF within 1 year after discharge [63], has a negative impact on patients’ quality of life, as well as on the healthcare systems. Therefore, it appears crucial to develop efficient tools in order to predict patient’s re-hospitalization probability and related causes of readmission. This would primarily help tailor patients’ remote support and education after discharge. Also, the early identification of patients at higher risk would improve the scheduling of potentially life-saving follow-ups. Accordingly, several works focused on this problem, exploring the use of DL methods applied to different types of data. Those studies are discussed below, and summarized in Table 3.

**Table 3:** Summary of recent studies exploiting deep learning algorithms for predicting HF readmission after discharge

Author	Year	Outcome	Data source	Dataset	Algorithm	Results
Xiao et al. [64•]	2018	30-day hospital readmission prediction	EHRs	5393 congestive HF patients	RNN with gated recurrent unit	AUC: 61.03% PR-AUC: 38.94% Accuracy: 69.34%
Awan et al. [65•]	2019	30-day readmission prediction or death	Linked administrative health dataset	10,757 over-65 HF patients	Multi-layer perceptron ANN	AUC: 62.8% PR-AUC: 46.1% Accuracy: 64.93% Sensitivity: 48.42% Specificity: 70.01%
Allam et al. [66•]	2019	30-day readmission prediction	Hospital claims dataset	272,778 patients, 343,328 HF admissions	Several deep learning models were tested. RNN combined with conditional random fields outperformed the others	AUC: 64.2%
Golas et al. [48•]	2018	30-day readmission prediction	EMRs	11,510 HF patients	Deep unified network	AUC: 70.5% Accuracy: 76.4%
Chen et al. [40•]	2020	1-year readmission prediction	EHRs	736 heart failure	Attention-based neural network	AUC: 69.1% Accuracy: 66.7% F1: 74.9% Recall: 79.5% Precision: 71%
Koehler et al. [73••]	2018	Compare non-invasive multi-parameter remote monitoring of HF patients with usual care to identify patients at higher risk	Body weight, blood pressure, electrocardiogram, heart rate, peripheral capillary oxygen saturation, self-rated score of the health status	796 patients assigned to the remote monitoring group and 775 to the control group	Fontane software (T-Systems International GmbH, Frankfurt, Germany), integrates business intelligence algorithms	Percentage of days lost: 4.88% in the remote patient management group and 6.64% in the usual care group (*p* = 0.0460) All-cause death rate (100 person-years of follow-up): 7.86 in the remote patient management group compared with 11.34 in the usual care group (*p* = 0·0280)
Gontarska et al. [74••]	2021	Risk score prediction	Age, weight, blood pressure, oxygen saturation, gender, diabetes, NYHA class, symptoms and signs of heart failure, ECG-extracted heart rate, sinus rhythm, ventricular tachycardia, atrial fibrillation, self-assessed state of health, weight difference, social variables	763 patients	Deep neural network	AUC: 84% PR-AUC: 19%
Stehlik et al. [76••]	2020		Wearable multi-parameter sensor: heart rate and its variability, arrhythmia burden, respiratory rate, physical activity, body posture	100 patients	Similarity-based machine learning algorithms	10-day window: AUC: 85.7% (HF hospitalization), 80.4% (unplanned non-trauma hospitalization) Event-specific: AUC: 89.3% (HF hospitalization), 83.6% (unplanned non-trauma hospitalization)

*HF*, heart failure; *HERs*, electronic health records; *EMR*, electronic medical records; *ECG*, electrocardiography; *NYHA*, New York Heart Association; *AUC*, area under curve; *PR-AUC*, area under the precision-recall curve; *ANN*, artificial neural network; *RNN*, recurrent neural network

Since discharge, e-Health solutions could support the prediction of patient’s outcome and probability of re-hospitalization. Among these, EHRs contain a huge amount of data, ranging from anthropometrics and demographic to prescribed therapies, comorbidities, and vital signs. Several works in the literature examined the possibility to apply DL models to EHRs in order to make accurate predictions of hospital readmissions in HF patients. An example of the increasing interest towards this field is represented by CONTENT [64•], a DL model based on a RNN with gated recurrent unit aiming at predicting 30-day hospital readmissions. It was developed using the EHRs of 5393 congestive HF patients, embedding data relevant to patients’ diseases, laboratory tests, and medications. Although outperforming other existing models, the results obtained in this work remain unsatisfactory, with 38.94% mean precision-recall AUC, 61.03% receiver operating characteristic AUC, and 69.34% accuracy. Similar results were also obtained with a multi-layer perceptron ANN applied to a linked administrative health dataset (10,757 over-65 HF patients) obtained from the Western Australian Data Linkage System [65•], as well as with a RNN combined with conditional random fields applied to a large hospital claims dataset [66•]. A deep unified network model developed on data obtained during inpatient and outpatient visits provided slightly improved results, with mean AUC equal to 70.5% and an accuracy of 76.4% in predicting 30-day readmission in HF patients [48•].

A key characteristic for a successful introduction of an AI model in the clinical practice stands in its interpretability, thus generally resulting in a higher propensity towards ML compared to DL techniques. Attention-based neural network prediction models represent a valid solution. A recent study [40•] evaluated the possibility to predict all-cause readmission in HF patients within 1 year after discharge using an attention-based neural network built on data contained in the EHRs of 736 HF patients. The proposed model assigns to each feature an “attention weight” indicating its importance in predicting readmission, and thus supporting clinicians in identifying patients at higher risk of a forthcoming relapse. For example, the analysis of the levels of B-type natriuretic peptide is widely used in clinical practice for the diagnosis of HF. As expected, this clinical feature was associated with considerably higher attention rates in the majority of the patients compared to the other features. Results appeared promising, although the achieved statistics, including mean F1-score and AUC values, remained below 80%, and thus requiring further improvements.

The application of interpretable DL methods could also take advantage of big data coming from EHRs in order to characterize subtypes of HF patients. An example can be observed in the study of Xiao and colleagues [64•], in which the authors were able to identify 20 subgroups of congestive HF patients, each possibly exhibiting different comorbidities that could impact the progression of this syndrome and consequently the readmission risk [3]. This approach could pave the way towards the identification and development of personalized and targeted home-care pathways.

In this context, remote monitoring solutions could effectively support HF patients in managing their condition and improving their quality of life, thus reducing the risk of readmission and mortality [67, 68]. Current methods primarily include telemonitoring with implantable devices, such as in the CardioMems [69] and the IN-TIME approach [70], which are recommended as Class II for use in selected patients by the 2016 ESC guidelines for the diagnosis and treatment of acute and chronic HF [3]. Thanks to the advances in technology and communication systems, non-invasive telemedicine solutions, including telephone-based monitoring and education, wearable and mobile health, have been implemented and tested, appearing particularly promising for patients that are not assigned to an implanted monitoring approach.

For example, remote monitoring of body weight is recommended in HF patients. However, daily monitoring of this parameter alone showed no evidence in identifying higher risk patients [71, 72]. A successful example of non-invasive multi-parametric remote patient monitoring is represented by the TIM-HF2 (Telemedical Interventional Management in Heart Failure II) prospective randomized controlled trial, with 796 patients assigned to the remote monitoring group and 775 to the control group [73••]. The study involved the daily measurement and transmission of several physiological parameters, including body weight, blood pressure, electrocardiogram, heart rate, and peripheral capillary oxygen saturation, as well as a self-rated score of the health status, fostering the cooperation of the telemedical center with cardiologists and general practitioners. The collected data were analyzed using the CE-marked Fontane telemedicine software (T-Systems International GmbH, Frankfurt, Germany), which integrates scalable business intelligence methods in order to assign a patient to a risk category [67]. Results showed that this approach effectively supported the identification of higher risk patients, accelerating tailored intervention and consequently reducing days lost during 1 year of follow-up and all-cause mortality. A recent study further improved these results, implementing a DL neural network model based on the TIM-HF2 database, which allowed to reach a mean AUC value of 84% [74••].

Wearable devices could additionally promote the continuous monitoring of patients’ health after discharge, thus representing an opportunity to improve remote monitoring and healthcare [75]. The LINK-HF study aimed at evaluating the accuracy of predicting deterioration which leads to re-hospitalization in HF patients using a wearable sensor (Vital Connect, San Jose CA) worn on the chest [76••]. Of note, this device recorded continuous acquisition of ECG, accelerometric signal, skin impedance, and skin temperature, thus permitting the monitoring of heart rate and its variability, arrhythmia burden, respiratory rate, physical activity, and body posture. Collected data were streamed to a smartphone and analyzed in Cloud. Similarity-based ML algorithms were able to generate a multivariate index which indicates the level of change of the acquired vital parameters. The presented platform appeared successful in predicting patient’s readmission due to worsening HF with a sensitivity from 76.0 to 87.5% at a specificity level of 85%.

## Challenges for Deep Learning

DL has demonstrated promising results with better performance in HF evaluation compared to ML and conventional algorithms, which could not be expected a priori. As DL algorithms require larger amount of data in order to provide high-quality results, this may limit their development especially in a clinical context, considering that the labeling data procedure is a time-consuming and tedious task for expert clinicians. Moreover, normal cases are often predominant over pathological ones, leading to unbalanced datasets which may originate biased predictions.

In contrast to conventional diagnostic and prognostic models, and similarly to ML, DL does not assume linear relationship among variables, leading to a better patient-level therapy treatment decisions. In some clinical trials, DL provided performance comparable to those of statistical linear models as logistic regression, suggesting how, depending on the type of data, a different analysis might be more suitable. Specifically, future studies could facilitate the integration of ML/DL models with statistical classifiers.

In addition, DL application in healthcare poses more challenges because data are often highly heterogeneous, noisy, and incomplete, and the number of available patients is usually limited, thus complicating the proper convergence of the DL algorithm and reliability of the results (i.e., garbage in results in garbage out). Moreover, the repeatability of the performance obtained with supervised models trained on specific datasets (i.e., monocentric, or obtained using the same equipment) onto data collected within other centers as well as with other equipment, or with different underlying patient factors (i.e., gender distribution, ethnicity, morbidities), needs to be further validated to avoid introducing biases in the results. Therefore, a standardized framework on how to perform and validate clinical studies would be required before implementation of DL into routine clinical use. Indeed, the impact of DL on the clinical decision-making process, on resources utilization and on value-based practice, has not been yet properly investigated. Moreover, the current literature reports an unbalance distribution of studies between ML and DL, with a limited number of DL studies, probably due to the limited availability of data. Indeed, in [77], the authors suggest that a substantially investment will be required in order to create high-quality annotated datasets for the development and the success of DL methods.

Another main limitation of DL models is inherent to the limited explicability of their results in a way that clinicians could understand. Opposite to ML, as the features are determined by the network itself, without a relation with possible features that a human could extract (i.e., mean, standard deviation, common parameters in the temporal or in the frequency domain), often it is not possible to understand which parameters and why have contributed to the generated output. This is particularly critical for decision support systems, where there is the need for the physician to comprehend and evaluate the source of the suggested action before taking the final decision and associated responsibility. Also in other fields, ethical issues have been raised concerning poor explicability, possibly leading to severe consequences [78, 79]. This condition of non-interpretability collides against the concept of evidence-based medicine, the cornerstone for clinical applications of DL, thus potentially limiting its utilization into clinical practice [80]. Possible solutions to cope with this limitation consist in the introduction of attention-base explainable DL methods, where the network is forced to learn on pre-defined attention maps on the original data, that can be visualized to better understand the origin of its results.

An additional aspect that could limit diffusion of DL in the medical field concerns the need for clinical assessment related to the software certification as medical device, as currently regulated by the EU legislation [81]. In fact, such software must undergo approval by notified bodies before being introduced in clinical practice, and proper accuracy and increased benefit over risk need to be demonstrated a priori. Additionally, as these networks are currently evolving based on the constant availability of data, the problem of re-certification over time has been posed to verify the same longitudinal performance.

## Conclusion

It is evident from the literature that DL algorithms have witnessed increasing applications in different aspects of the management of HF patients, with the aim to improve efficiency in diagnosis and prognosis. These methods have already demonstrated to overcome the performance of conventional approaches in different clinical setting, being able to integrate different data sources in order to improve diagnosis and prediction, potentially leading to tailored treatments.

The results described in this review have illustrated the potential capabilities of DL methods to improve prediction relevant to mortality and hospital readmission, highlighting how these promising tools could introduce substantial positive and significant changes in the clinical workflow in the future treatment of HF in the near future. For example, the increasing availability of smart analysis in EHRs based on DL applications will reduce the need for scoring systems, enabling personalized treatment for HF patients. DL analytical skills have been shown to be superior to those of expert clinicians, since humans can handle only a limited number of cognitive information (i.e., variables in structure data) at once [82, 83], thus facilitating clinical support for early HF risk identification. With a rapidly growing scenario in cardiovascular medicine, DL has the potential of paving the way towards a new generation of predictive methods in healthcare that could automatize essential processes involved in treatment planning, helping in identifying hidden information in complex and heterogeneous datasets to effectively support clinicians in their daily activities. In this scenario, DL has showed potential to classify HF patients into novel phenotypes who might benefit of specific treatments, as well as for early diagnosis of HF to improve its prognosis. The integration of different data sources including EHRs, genomics, and remote patient monitoring could provide a better description on the HF patient individual status, which might support clinicians regarding appropriate intervention and therapy, hospital discharge, and hospital re-admissions. However, for DL to become part of clinical practice, several ethical and regulatory issues need to be properly addressed and solved. These challenges introduce both new opportunities and the need of further research to provide more evidence about the effective benefit of these algorithms in being translated into better quality of care for patients, improved outcomes, and lower healthcare costs.
